# Matrix Metalloproteinases in Health and Disease

**DOI:** 10.3390/biom10081138

**Published:** 2020-08-01

**Authors:** Raffaele Serra

**Affiliations:** Department of Medical and Surgical Sciences, University Magna Graecia of Catanzaro, Viale Europa, Catanzaro, 88100 Germaneto, Italy; rserra@unicz.it; Tel.: +393-387-078-043

**Keywords:** matrix metalloproteinases, health, disease

Matrix metalloproteinases (MMPs) are members of an enzyme family and, under normal physiological conditions, are critical for maintaining tissue allostasis. MMPs can catalyze the normal turnover of the extracellular matrix (ECM) and its activity is also regulated by a group of endogenous proteins called tissue inhibitors of metalloproteinases (TIMPs) or other proteins, such as Neutrophil Gelatinase-Associated Lipocalin (NGAL). An imbalance in the expression or activity of the aforementioned proteins can also have important consequences in several diseases, such as cancer, cardiovascular disease, peripheral vascular disease, inflammatory disease, and others. In recent years, MMPs have been found to have an important role in the field of precision medicine as they may serve as biomarkers that may predict an individual’s disease predisposition, state, or progression. MMPs are also thought to be a sensible target for molecular therapy [1,2,3,4].

This Special Issue includes ten papers: seven original articles and three review articles dealing with a broad range of diseases related to MMPs.

The article by Santiago Ruiz et al [5] showed that several polymorphisms and genes associated with metalloproteinases influence the development of Hypersensitivity Pneumonitis (HP), an inflammatory disease caused by an exaggerated immune response to the inhalation of certain organic particles. Remodeling of the ECM in the airways and pulmonary interstice seem to relate to the worsening of lung function. This study documented that some polymorphisms in the MMP-1 and MMP-2 genes are associated with the risk of hypersensitivity pneumonitis, and, in particular, the MMP-2 polimorphism also correlates with lung function.

The study by Cione E et al. [6] evaluated the expression of MMP-2, MMP-9, and NGAL in the plasma and tissue of patients with aneurysmal disease. In particular, the modulation of these three biochemical indicators, related to vascular remodeling, was also studied in patients under statin treatment. The study deepens the pathophysiology of arterial aneurysms in light of ECM alterations, and suggests that statin treatment may have a role in the prevention of aneurysm growth and subsequent rupture by modulating the effects of MMP-2, MMP-9, and NGAL on ECM alterations, endothelial function, and also reducing inflammation and oxidative stress.

The paper by Rautava J et al. [7] deals with Crohn’s disease (CD) a complex inflammatory disease of the gastrointestinal tract, and the tendency of such patients to develop periodontitis, caries, and oral mucosal lesions. The study speculates that the dysregulation of the immune system in CD may have an effect on MMP-8 levels in the oral cavity. In this context, MMP-8 seems to be the key inflammatory mediator in these conditions; In fact, elevated MMP-8 levels have been detected in CD patients both in the intestine and in the oral cavity.

The article by Rodríguez-Sánchez E. et al. [8] explores the variations in circulating active MMP-9 levels during Renal Replacement Therapy (RRT), which is a condition that may be complicated by a chronic state of inflammation and a high mortality risk. The study documented that MMP-9 is an effective marker of vascular dysfunction in patients undergoing RRT.

The study by Heinzmann D et al. [9] explored the recruitment of leukocytes and platelets to activated endothelia as well as platelet–leukocyte interactions in the context of thromboinflammatory mechanisms. In particular, this study highlights that the surface receptor CD147 (basigin, extracellular matrix–metalloproteinase inducer; EMMPRIN) has a role in the host defense from self-derived, as well as invading targets, and it is also a major factor modulating the expression of MMPs. In this context, CD147 seems to have pathophysiological relevance in platelet–leukocyte interactions in thrombosis related mechanisms.

The paper by Ferrigno A et al. [10] studied the involvement of MMPs in hepatic ischemia/reperfusion (I/R) injury. This study showed the precise role of MMPs, in particular MMP-2 and MMP-9, that may contribute to the development of organ dysfunction and injury, especially in the early phase of this condition.

The article by O’Sullivan S. et al. [11] studied the role of MMP-9 in inflammatory bowel disease (IBD) and investigated the mechanism of action of barbiturate-nitrate hybrid compounds and their component parts using models of intestinal inflammation in vitro in order to inhibit the upregulation of MMP-9 gene expression. This study highlights the potential of treating colonic inflammation by means of downregulating MMP-9 activity, and subsequent inflammatory sprout in IBD.

The study by Provenzano M. et al. [12] aimed to examine the role of MMPs in increasing the risk of peripheral vascular disease (PVD) by the specific factors related to Chronic Kidney Disease (CKD). This paper speculates on the possibility of a strict link between PAD and PVD, mediated by MMPs, in particular MMP-2 and MMP-9, and the latter also sustained by an increase in NGAL circulating levels that are also known to be directly related to diabetic status and inversely to estimated glomerular filtration rate (eGFR) levels.

The paper by Laronha H. [13] extensively reviewed the currently reported synthetic inhibitors of MMPs and also provided an accurate description of their properties. In particular, Hydroxamate-Based Inhibitors, Non-Hydroxamate-Based Inhibitors, Catalytic Domain (Non-Zinc Binding) Inhibitors, Allosteric and Exosite Inhibitors, and Antibody-Based Inhibitors are presented and discussed.

The article by Liu Z. et al. [14] reviewed the expression, regulation, novel substrates, and mechanisms of MMP-7 in several kidney diseases. In particular, MMP-7 was found upregulated in acute kidney injury (AKI), CKD, and glomerular diseases and was also predominantly localized in renal tubular epithelia. Furthermore, MMP-7 levels may serve as a noninvasive biomarker for predicting AKI prognosis and monitoring CKD progression.

This Special Issue describes important findings related to MMPs function, and dysregulation in several areas, such as vascular, kidney, and respiratory systems and also highlights the most recent progress on the knowledge and the clinical and pharmacological applications related to the most relevant areas of healthcare.
